# Assessment of Quality and Efficiency of Cold-Pressed Oil from Selected Oilseeds

**DOI:** 10.3390/foods12193636

**Published:** 2023-09-30

**Authors:** Abraham Kabutey, David Herák, Čestmír Mizera

**Affiliations:** Department of Mechanical Engineering, Faculty of Engineering, Czech University of Life Sciences Prague, 165 20 Prague, Czech Republic; herak@tf.czu.cz (D.H.); mizera@tf.czu.cz (Č.M.)

**Keywords:** edible oilseeds, screw-pressing, oil yield, seedcake sediments, physicochemical properties

## Abstract

In this present study, an oil press was used to process 200 g each of sesame, pumpkin, flax, milk thistle, hemp and cumin oilseeds in order to evaluate the amount of oil yield, seedcake, sediments and material losses (oil and sediments). Sesame produced the highest oil yield at 30.60 ± 1.69%, followed by flax (27.73 ± 0.52%), hemp (20.31 ± 0.11%), milk thistle (14.46 ± 0.51%) and pumpkin (13.37 ± 0.35%). Cumin seeds produced the lowest oil yield at 3.46 ± 0.15%. The percentage of sediments in the oil, seedcake and material losses for sesame were 5.15 ± 0.09%, 60.99 ± 0.04% and 3.27 ± 1.56%. Sediments in the oil decreased over longer storage periods, thereby increasing the percentage oil yield. Pumpkin oil had the highest peroxide value at 18.45 ± 0.53 meq O_2_/kg oil, an acid value of 11.21 ± 0.24 mg KOH/g oil, free fatty acid content of 5.60 ± 0.12 mg KOH/g oil and iodine value of 14.49 ± 0.16 g l/100 g. The univariate ANOVA of the quality parameters against the oilseed type was statistically significant (*p*-value < 0.05), except for the iodine value, which was not statistically significant (*p*-value > 0.05). Future studies should analyze the temperature generation, oil recovery efficiency, percentage of residual oil in the seedcake and specific energy consumption of different oilseeds processed using small-large scale presses.

## 1. Introduction

In the literature, several of our published studies have been primarily focused on the mechanical and relaxation behaviours of bulk oilseeds under the uniaxial compression process [1,2,3,4,5,6,7,8,9]. In these studies, the effects of processing conditions such as heating temperatures, freezing temperatures, moisture content, pressure, compression speed and diameter of pressing vessels on force–deformation curves, hardness, energy demand, volume energy, oil-point pressure and oil yield were reported. This information is useful for improving the performance of the mechanical screw-pressing of oilseeds as it achieves a higher percentage of oil recovery, reducing the residual oil in the seedcake and ensuring efficient energy utilization.

Ongoing research is needed to better understand the oilseeds’ flow and expression in a screw press [10]. Screw presses possess capacities between 9 and 16,000 kg/h and are commonly used for vegetable oil extraction from various oilseeds such as flax, jatropha, linseed, canola, crambe and chia seeds. Smaller-capacity presses are often employed in rural areas, whereas the larger presses are operated in large-scale industry [11,12,13,14,15,16]. Several factors, including pressure, temperature, screw rotation speed, the diameter of the restriction die, moisture content and pressing time, contribute to a lower-percentage oil yield, thus leaving larger amounts of residual oil, about 8 to 14%, in the press cake. Pre-treatment of the seeds and adjustments to the screw press configuration and components have enhanced oil yield recovery [17,18,19,20]. In the industry, however, pre-pressing and solvent extraction steps are combined to achieve a high oil yield with residual oil content lower than 3% [17,18]. Generally, screw press operation requires an increase in temperature in the barrel to achieve a high oil extraction yield, but an increase in temperature has been reported to affect the chemical and sensorial qualities of the oil during extraction [21,22]. 

Cold-pressing is a cost-effective method, safe for consumers and the environment in terms of the absence of chemicals and refining processes [16,19,23,24,25]. Obtaining high-quality edible oils using screw presses with or without any pre-treatments requires an in-depth analysis of the screw-pressing operation by processing different oilseeds simultaneously [17,18,26]. This information, however, has not been adequately reported in the literature. The objectives of the present study were to: determine the percentage amounts of oil yield, seedcake, sediments in the oil and material losses (oil and sediments); analyze the effect of storage time on seedcake sediments in the oil and determine the quality parameters (moisture content, peroxide value, acid value, free fatty acid content and iodine value) of the extracted oils of sesame, pumpkin, flax, milk thistle, hemp and cumin seeds. In addition, a brief literature background of the selected oilseeds was provided. 

## 2. Background of Selected Oilseeds

### 2.1. Sesame

Sesame (*Sesamum indicum* L.) is believed to originate from the savanna of central Africa, and it has been extensively cultivated in Asia and Africa [27], with India, China, Sudan and Burma accounting for 60% of the world’s sesame production [28]. It is approximately 37–63% oil, which is mainly composed of mono and polyunsaturated fatty acids, accounting for almost 85% of the total fatty acids [29]. Sesame is rich in lipids (44–58%), protein (10–25%) and carbohydrates (3–20%) [30]. It also contains significant amounts of phytosterols, tocopherols and lignans [27,31]. 

### 2.2. Pumpkin

Pumpkin (*Curcurbita pepo*) is a tropical and subtropical oilseed crop which is cultivated particularly for consumption and medicinal purposes [32,33]. Important species of pumpkin include *Cucurbita pepo, Cucurbita maxima*, *Cucurbita ficifolia,* and *Cucurbita stilbo* [34]. Its oil content ranges from 22 to 64% [8,35,36,37]. Pumpkin seeds have a high nutritional content: proteins (24–40%), carbohydrates (12–14%), lipids (44–52%) and fibre (4–6%), depending on the variety [38,39]. Experimental studies have demonstrated the anti-microbial, anti-diabetic, anti-hyperlipidemic, anti-carcinogenic, anti-hypertensive, anti-inflammatory, anti-depressant, anti-oxidant and anti-helminthic effects of pumpkin seeds [34,40,41,42].

### 2.3. Flax

Flax (*Linum usitatissimum* L.), also known as common flax or linseed, is widely grown in temperate regions of the world [42,43]. The major producers of flax are Canada, Russia, China, Kazakhstan and the United States of America [42]. It is an important source of industrial vegetable oil and has several commercial, and medicinal values and other functional properties [43,44]. It is 38–44% oil, which is rich in omega-3 fatty acids and linolenic acid; 1–5% lignans, associated with high anti-oxidant activity; 10–31% proteins; and about 28% dietary fibre [45,46,47]. Applications of the oil include human consumption, animal feed and the manufacturing of paints and dyes [47,48]. Some of its health benefits include a reduction in hypertension, serum triglycerides and cholesterol, anti-inflammatory action and anti-cancer activity [46,49].

### 2.4. Milk Thistle

Milk thistle (*Silybum marianum* L.) belongs to the *Asteracae* flowering plant family [42]. The flowers are purple and the leaves are dark green with characteristic spiny edges; the leaves exude milk sap. The plant originated in the Mediterranean, and it is now grown in Europe, Asia, and northern Africa [50]. The main active substance in *Silybum marianum* is silymarin, composed of silybin, isosilybin, silydianin and silychristine [51,52]. The fruit/seed is 20–30% oil and 25–30% protein and has a high copper content (17 mg/g) [42]. The oil is a by-product of silymarin, which is rich in essential fatty acids, phospholipids, sterols and vitamin E [51,53]. The roots, flowers, leaves and stalks are used as forage for animals [54]. The plant is used as a remedy for liver diseases [55,56], and it is being studied for its hepatoprotective, neuroprotective, nephroprotective and cardioprotective functions [42,52,57,58].

### 2.5. Hemp

Hemp (*Cannabis sativa* L.) is an annual herb with edible and medicinal properties [42,59,60,61]. The plant can adapt and respond to climatic changes, and it can be found in different forms based on genetic variability [62]. Presently, the nutritional uses of hemp have been concentrated on its seeds, which are rich in oil and protein [61]. Hemp seed oil contains roughly 25–38% oil, which is usually obtained via cold pressing, allowing for a larger amount of minor compounds to be extracted with the oil [63,64,65,66]. The protein and carbohydrate contents range from 20–25% and 20–30%, respectively [63]. The presence of minor anti-oxidant components, such as tocopherols, increases the nutritional value, the health properties of hemp seed oil and its shelf-life [66,67]. Hemp seed oil quality should be guaranteed for consumers by setting specific parameters because of its differentiation due to several factors such as seed variety and extraction methods [62].

### 2.6. Cumin

Cumin (*Cuminum cyminum L.*) seeds are a highly appreciated spice with several applications, reaching global consumption of around 187,000 metric tonnes [42,68]. They are widely cultivated in Uzbekistan, Tajikistan, Turkey, Morrocco, Egypt, India, Syria, Mexico, Pakistan and Chile [69,70]. The seeds have a distinctive aromatic odour and a spicy, faintly pungent flavour. An essential oil content of cumin seeds between 1.16 and 1.98% has been reported [68,71]. Under optimal conditions, the highest essential oil yield of cumin seeds has been determined to be 2.22% [72]. In the pharmaceutical industry, cumin components are associated with anti-cancer, anti-inflammatory, anti-spasmodic, detoxifying, anti-microbial, diuretic, carminative and anti-oxidant properties [68,73,74,75].

## 3. Materials and Methods

### 3.1. Selected Oilseeds Samples

In this study, the selected oilseeds were sesame (white/natural type), pumpkin (white type—unhulled), flax (brown type), milk thistle (dark–brown type—unhulled), hemp (brown type—unhulled) and cumin (brown type). The selection procedure was carried out based on a background search in the literature and the scope of our ongoing project. The oilseed samples were purchased from Stredni, Prague, Czech Republic. They were properly sealed and kept in laboratory conditions at a temperature of 20.07 ± 0.95 °C and humidity of 43 ± 1%.

### 3.2. Determination of Moisture Content of Selected Oilseeds

The moisture content of the selected oilseeds was determined using the hot-air oven method by drying the samples at 105 °C for 17 h [76,77]. The calculation was carried out using Equation (1) [9,78] as follows:

(1) $$MC=\left[\left(\frac{m_{b}-m_{a}}{m_{b}}\right)\cdot 100\right]$$

where MC is the percentage sample moisture content (% w.b.), and $m_{a}$ and $m_{b}$ are the masses of the samples before and after oven drying (g).

### 3.3. Oil Processing from Selected Oilseeds

**Figure 1:**
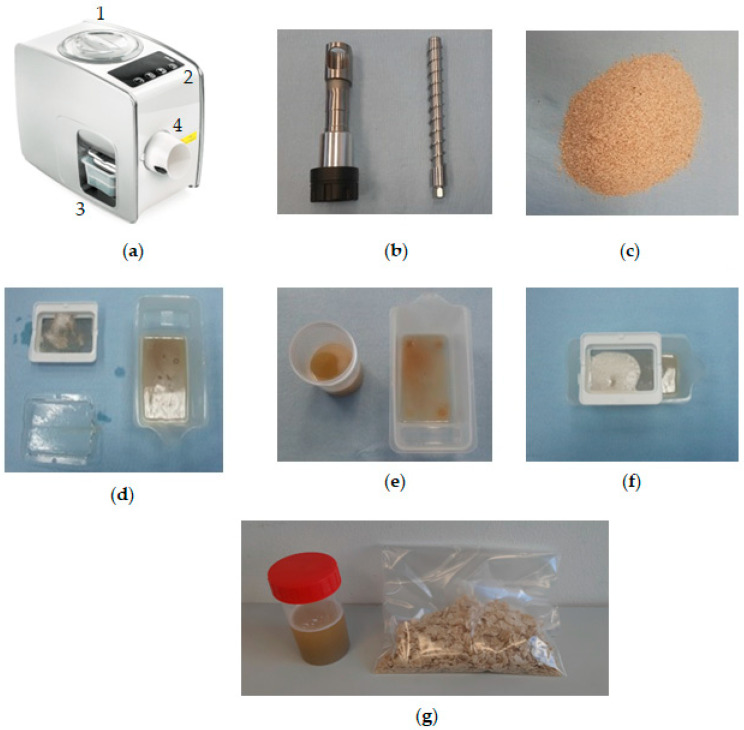
Oil extraction process of sesame similar to other oilseeds: (**a**) oil press machine, 1: hopper; 2: oilseed selection program; 3: oil flow chamber with press components; and 4: seedcake exit and removal of screw shaft and casing for cleaning; (**b**) screw shaft and casing; (**c**) sesame sample; (**d**–**f**) press components, and (**g**) extracted crude sesame oil in a small container for storage and seedcake in a transparent plastic bag.

The selected oilseeds (sesame, pumpkin, flax, milk thistle, hemp and cumin) were processed for their crude oils using a Yoda Electric Oil Press (Model: YDZY02A1/YDZY02A2/YDZY02A3; Naarden, The Netherlands) (Figure 1a). The weight of the press is 6.8 kg. The components of the oil press are the screw shaft and barrel/casing and a rectangular plastic beaker for oil collection with a filter/sieve. The filter ensures that clean oil is obtained with minimal seedcake sediments. The screw shaft dimensions are as follows: total shaft length: 197.53 mm, thread length: 162.32 mm, core diameter: 11.85 mm, thread diameter: 19.97 mm, thread pitch: 11.97 mm, thread thickness: 2.39 mm, length of screw shaft casing: 183.44 mm and end diameter: 25.54 mm. The oil press is powered by an electric motor with a voltage rating of 220-240V/50 HZ. The motor power is 180 W and the heating power is 330 W. There are seven modes of oilseed selection for processing. The temperature of the extracted oil does not exceed 40 °C. Although a cold-pressed oil can be obtained, the screw shaft can generate a higher temperature depending on the hardness of the oil-bearing material and the preset program that goes with it during processing [79]. Before the oil extraction, the press was allowed to pre-heat for about 30 s to 1 min depending on the oilseed type. The crude oil was extracted from 200 g of each of the selected oilseed samples, which were fed through the press hopper (diameter 125.94 mm and depth 100 mm). The crushing of the seeds is carried out by the rotating screw inside the barrel. The crude oil and the seedcake are obtained simultaneously via the oil output chamber and seedcake exit. The recorded extracted time for all the samples was approximately 8 min.

**Figure 2:**
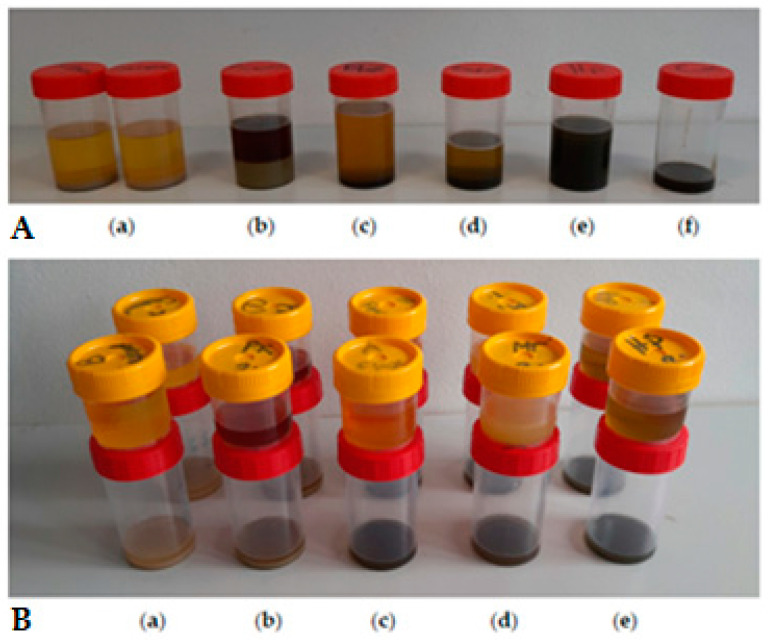
(**A**) Extracted oils with seedcake sediments and (**B**) separated oils and sediments from (**a**) sesame; (**b**) pumpkin; (**c**) flax; (**d**) milk thistle; (**e**) hemp and (**f**) cumin.

**Figure 3:**
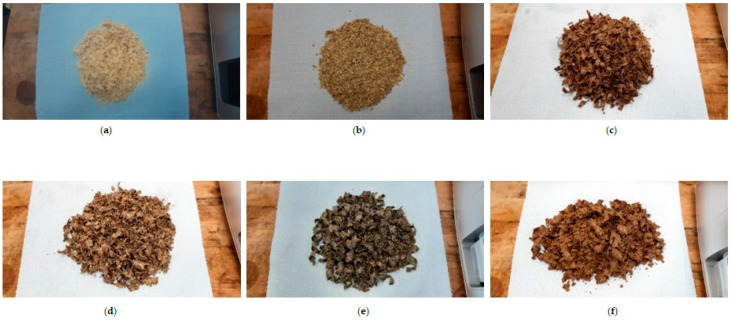
De-oiled seedcakes from (**a**) sesame; (**b**) pumpkin; (**c**) flax; (**d**) milk thistle; (**e**) hemp and (**f**) cumin.

It is important to mention that the oil press can be operated continuously for 45 min. Afterwards, it is necessary to cool the press for 30 min before further operation. In addition, for screw press operation, continuous transport of the oilseeds via the screw shaft causes pressure to increase, which increases friction inside the screw press. This generates heat, which lowers the viscosity of the oil, thereby increasing the oil flow rate [79]. The extracted crude oil and seedcake were weighed for further calculations. Figure 2A,B show the extracted crude oil (with seedcake sediments) and refined oil (without seedcake sediments). The de-oiled seedcakes from each sample are shown in Figure 3.

### 3.4. Calculation of Oil Yield and De-oilseed Seedcakes

The percentage oil yield was calculated using Equation (2) [77,80,81] as follows:

(2) $$O_{YD}=\left[\frac{M_{OL}}{M_{SP}}\right]\cdot 100$$

where $O_{YD}$ is percentage oil yield (%), and $M_{OL}$ is the mass of oil obtained as the difference between the mass of the seedcake and the initial mass of the sample $M_{SP}$ (g). The mass of the seedcakes (g) was determined as the difference between the mass of the initial sample and the mass of the extracted crude oil, which was calculated as a percentage.

### 3.5. Evaluation of Oil Output and Quality

The extracted crude oils (oil and sediments) as shown in Figure 2I were left in the laboratory for 21 days in storage to allow the sediments to settle completely at the bottom of the container. Every 7 days, a pipette filler bulb was used to syphon off or recover the cleaned oil into a separate container without any sediments, as shown in Figure 2II. The cleaned oils obtained during the 7 days of storage were used for quality assessment in terms of peroxide value, *PV* (meq O_2_/kg oil), acid value, *AV* (mg KOH/g oil), free fatty acid, *FFA* (mg KOH/g oil) and iodine value, *IV* (g l/100 g), via a titration technique following AOAC procedures [7,82]. The cumin seed oil was exempted from the quality evaluation due to low oil output and the difficulty of separating the seedcake sediments from the oil (Figure 2If). The *PV*, *AV*, *FFA* and *IV* were determined using Equations (3) to (6) [83,84,85] as follows:

(3) $$PV=\frac{V_{T}\cdot N_{T}\cdot 1000}{W}$$

(4) $$AV=\frac{V_{T}\cdot N_{T}\cdot 56.1}{W}$$

(5) $$FFA=\frac{AV}{2}$$

(6) $$IV=\frac{V_{T}\cdot N_{T}\cdot 12.69}{W}$$

where $V_{T}$ is the volume of the titrant used for the titration (for *PV*, Sodium thiosulphate: Na_2_S_2_O_3_ and for *AV*, Potassium Hydroxide: KOH) in mL, $N_{T}$ is the normality of the titrant used for the titration, *W* is the weight of the oil sample in grams, and 56.1 and 12.69 represent the molecular weight of KOH and Iodine.

### 3.6. Statistical Analysis

All the experiments were duplicated. Descriptive statistics (mean and standard deviation) and ANOVA were used to interpret the data at 0.05 level of significance using Statistica 13 software [86].

## 4. Results and Discussion

**Figure 4:**
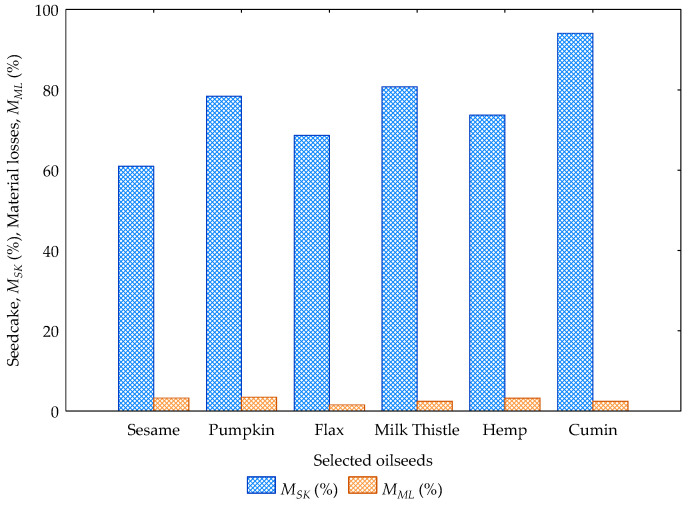
Comparison of the percentage of seedcake $M_{SK}$ and material losses $M_{ML}$of the selected oilseeds.

**Figure 5:**
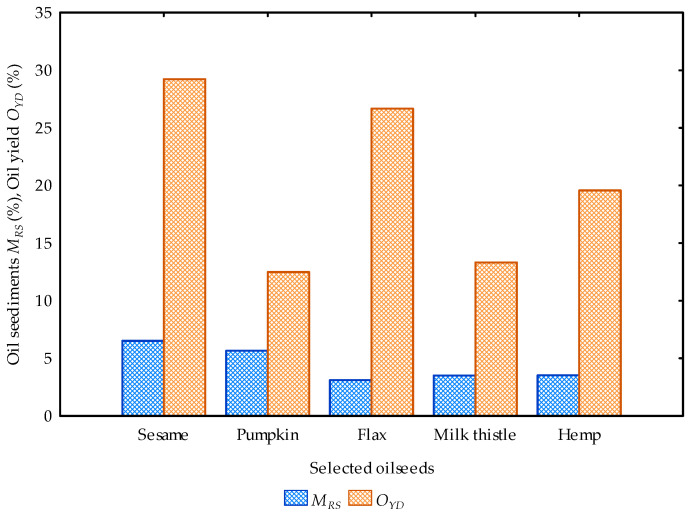
Comparison of the percentage seedcake sediments in the oil and oil yield of the selected oilseeds.

**Figure 6:**
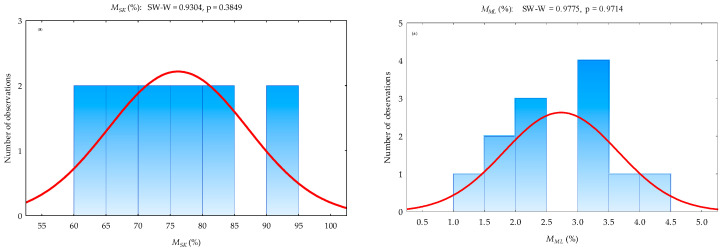

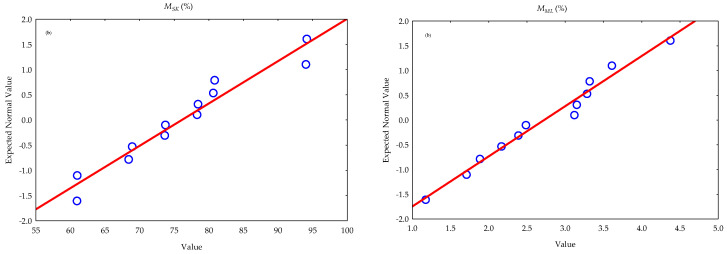
Test of normality: (**a**) histogram and (**b**) normal probability plot of the percentage seedcake ($M_{SK})$ and material losses ${(M}_{ML})$.

**Figure 7:**
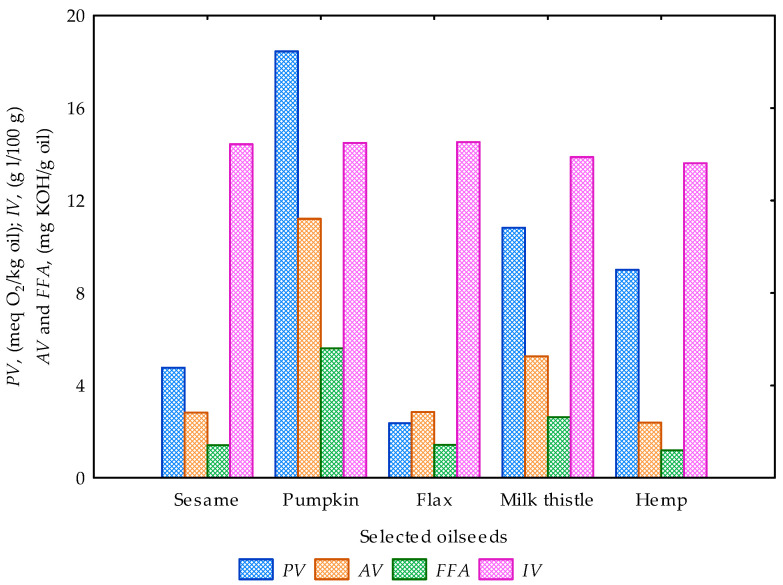
Comparison of the quality parameters of the cold-pressed oils from the selected oilseeds.

The characteristics of the crude oils extracted from the selected oilseeds are presented in Table 1, Table 2, Table 3, Table 4, Table 5, Table 6, Table 7 and Table 8 and Figure 4, Figure 5, Figure 6 and Figure 7. Firstly, the moisture content of the selected oilseeds ranged from 6.01 ± 0.13 to 9.38 ± 0.08% (w.b). as presented in Table 1. Pumpkin seeds had the lowest moisture content, whereas cumin seeds had the highest value. During the oil extraction process, moisture acts as a lubricant. Lower moisture content results in more sediments in the oil and causes higher frictional resistance, resulting in a higher pressing rate. Choking/jamming of the seeds or seedcake occurs with lower seed moisture content. In contrast, higher moisture content increases plasticity, thereby reducing the level of compression and contributing to poor oil recovery [87,88,89]. Singh and Bargale [11] found that the maximum oil recovery of rapeseed, 90.2%, was achieved at a moisture content of 7.5% (w.b). In most cases, the optimum moisture content varies among oilseeds and ranges from 2.1 to 11.5% (d.b.) [15,88,89,90]. For instance, Gaber et al. [89] indicated a higher oil yield of canola seeds at moisture contents between 5 and 11.5% (d.b.). However, at a higher moisture content of >11.5% (d.b.), the net oil yield was significantly reduced. Notably, an increase in moisture content leads to a decrease in oil yield [15,89]. The moisture content of seeds during the pressing process also affects the quality of oil. It has been reported that a decrease in moisture content causes an increase in chlorophyll and phospholipids contents in oil, whereas an increase in moisture content increases the sulfur, calcium and magnesium contents in oil [15,89,91]. Orhevba et al. [92] showed that moisture content had both increasing and decreasing effects on the quality parameters (saponification value, iodine value, free fatty acid and color) of neem seed kernel oil. The authors also observed that increasing the moisture content from 6.3 to 16.6% (w.b.) affected the color of the oil, which changed from brown to dark brown. Torres and Maestri [93] also reported that the color intensity of palm kernel and sesame oils increased with increasing moisture content.

Secondly, the determined parameters from each initial sample mass of 200 g were the mass of seedcake, the mass of oil and the mass of material losses (oil and sediments). The difference between the cumulative amounts of the mass of seedcake and the mass of oil and the initial sample mass represented the material losses. The extracted crude oils’ output data on the first day are shown in Table 1. The mass of the seedcake ranged from 121.97 ± 0.07 to 188.23 ± 0.15 g. The mass of oil ranged from 6.91 ± 0.29 to 71.49 ± 3.20 g, and the cumulative masses of seedcake and oil ranged from 193.07 ± 0.41 to 196.94 ± 1.00 g. The mass of material losses ranged from 3.06 ± 1.00 to 6.93 ± 0.41 g. The mass of oil included the seedcake sediments, which, in other words, can be described as the mass of crude oil. The material losses included the sediments on the sieve oil loss during the pressing operation and oil collection/transfer from one container to another. The effect of storage days on the extracted oils is presented in Table 2, Table 3 and Table 4. For the first 7 days, the mass of seedcake residues in the oil ranged from 9.38 ± 0.56 to 17.23 ± 0.49 g, whereas the mass of oil ranged from 21.86 ± 0.33 to 54.26 ± 3.69 g. The percentage values of seedcake sediments in the oil ranged from 4.69 ± 0.28 to 8.62 ± 0.25%, oil yield ranged from 10.93 ± 0.17 to 27.13 ± 1.85%, seedcake ranged from 60.99 ± 0.04 to 94.11 ± 0.07% and material losses ranged from 1.53 ± 0.50 to 3.47 ± 0.21%. After 14 days of storage, the mass of seedcake sediments ranged from 5.16 ± 0.64 to 11.60 ± 0.40 g, and the mass of oil ranged from 26.33 ± 0.54 to 59.89 ± 3.59 g. The percentage values of seedcake sediments and oil yield ranged from 2.58 ± 0.32 to 5.80 ± 0.20% and 13.17 ± 0.27 to 29.95 ± 1.80%, respectively. At 21 days of storage, the mass of seedcake sediments ranged from 4.12 ± 0.74 to 10.30 ± 0.18 g, and the mass of oil ranged from 26.75 ± 0.70 to 61.20 ± 3.37 g. The percentage values of seedcake sediments and oil yield ranged from 2.06 ± 0.35 to 5.15 ± 0.09% and 13.37 ± 0.35 to 30.60 ± 1.69%, respectively. For the cumin oil sample, the percentage of seedcake residues/sediments in the oil and the oil yield were not calculated during the storage periods due to cumin’s minimal oil output, which made it difficult to separate the oil from the seedcake sediments. Hence, cumin seeds proved unsuitable for processing under mechanical screw-pressing. As a result, cumin seeds obtained the highest seedcake result at 188.23 ± 0.15 g, with the lowest crude oil recovery rate at 6.91 ± 0.29 g, followed by milk thistle seeds, generating an amount of 161.54 ± 0.26 g with the second-lowest crude oil recovery at 33.64 ± 2.26 g, followed by pumpkin seeds, which produced an amount of 156.77 ± 0.15 g and the third-lowest crude oil recovery at 36.31 ± 0.26 g. Sesame produced the smallest amount at 121.97 ± 0.07 g, with the highest crude oil recovery at 71.49 ± 3.20 g. For all the storage periods, sesame seeds produced the highest amount of oil at 61.20 ± 3.37 g, representing a percentage oil yield of 30.60 ± 1.69%. This corresponds to a percentage seedcake sediment of 5.15 ± 0.09% and material losses of 3.27 ± 1.56%. Flax and hemp seeds followed, in that order, indicating that sesame, flax and hemp seeds contain more oil than milk thistle, pumpkin and cumin seeds do. Noticeably, sediments in the oil decreased with longer storage periods, thereby increasing the recovered oil or percentage oil yield.

Thirdly, based on the literature, the fat/oil content of sesame and pumpkin seeds ranges from 37–63% and 22–64%, respectively [8,29,35,36,37,38,42]. Flaxseed oil content ranges from 38–44% [45,46,47]. Milk thistle seed oil content ranges from 20–30% [49]. Hemp seed oil content ranges from 25–38% [63,64,65,66] and cumin seeds oil content ranges from 1.16–2.22% [68,72,73]. According to Hernandez-Santos et al. [94], the percentage of the oil yield relative to that of the oil content represents the oil expression efficiency. From this relation, the oil expression efficiency of the selected oilseeds was calculated for all samples except for cumin seeds. Sesame oil expression efficiency ranged from 48.57 to 82.70%, pumpkin: 20.89 to 60.77%, flax: 63.02 to 72.97%, milk thistle: 48.2 to 72.3% and hemp: 53.45 to 81.24%. To achieve high oil recovery efficiency, there is the need to use medium to large scale mechanical screw presses, coupled with advanced oil processing and refining techniques, to considerably reduce the residual oil in the seedcake, seedcake sediments in the oil and material losses (oil and sediments) during the pressing operation. The percentage of seedcake, material losses and sediments in the oil and oil yield are illustrated graphically in Figure 4 and Figure 5.

**Table 1:** Determined parameters of pressed oil from a one-day pressing operation.

Oilseeds	MC (% w.b.)	$M_{SP}$ (g)	$M_{SK}$ (g)	$M_{OL}$ (g) *	$M_{SK+OL}$ (g)	$M_{ML}$ (g) **
Sesame	7.96 ± 0.53	200	121.97 ± 0.07	71.49 ± 3.20	193.46 ± 3.13	6.54 ± 3.13
Pumpkin	6.01 ± 0.13	200	156.77 ± 0.15	36.31 ± 0.26	193.07 ± 0.41	6.93 ± 0.41
Flax	6.45 ± 0.16	200	137.37 ± 0.74	59.57 ± 1.74	196.94 ± 1.00	3.06 ± 1.00
Milk thistle	7.69 ± 0.14	200	161.54 ± 0.26	33.64 ± 2.26	195.17 ± 1.99	4.83 ± 1.99
Hemp	6.75 ± 0.06	200	147.37 ± 0.14	46.20 ± 0.33	193.57 ± 0.19	6.44 ± 0.19
Cumin	9.38 ± 0.08	200	188.23 ± 0.15	6.91 ± 0.29	195.13 ± 0.14	4.87 ± 0.14

MC: percentage sample moisture content (% w.b.); $M_{SP}$: mass of oilseed sample; $M_{SK}$: mass of seedcake; $M_{OL}$: mass of extracted crude oil; $M_{ML}$: mass of material losses (oil and sediments); *: with seedcake residues/sediments in the oil; **: seedcake residues/sediments on the sieve and oil loss via pressing and collection.

**Table 2:** Determined parameters of the pressed oil at 7 days in storage under laboratory conditions.

Oilseeds	$M_{RS}$ (g)	$M_{OL}$ (g) ***	$M_{RS}$ (%)	$O_{YD}$ (%)	$M_{SK}$ (%)	$M_{ML}$ (%)
Sesame	17.23 ± 0.49	54.26 ± 3.69	8.62 ± 0.25	27.13 ± 1.85	60.99 ± 0.04	3.27 ± 1.56
Pumpkin	14.45 ± 0.07	21.86 ± 0.33	7.23 ± 0.04	10.93 ± 0.17	78.38 ± 0.07	3.47 ± 0.21
Flax	9.38 ± 0.56	50.20 ± 1.18	4.69 ± 0.28	25.10 ± 0.59	68.69 ± 0.37	1.53 ± 0.50
Milk thistle	10.68 ± 1.44	22.96 ± 0.81	5.34 ± 0.72	11.48 ± 0.41	80.77 ± 0.13	2.42 ± 1.00
Hemp	9.49 ± 0.32	36.70 ± 0.01	4.75 ± 0.16	18.35 ± 0.01	73.69 ± 0.07	3.22 ± 0.10
Cumin	****	****	****	****	94.11 ± 0.07	2.43 ± 0.07

$M_{RS}$: mass of seedcake residues/sediments in oil; $M_{OL}$: mass of extracted crude oil; $O_{YD}$: oil yield; $M_{SK}$: mass of seedcake; $M_{ML}$: percentage material losses; ***: without seedcake residues/sediments in the oil and ****: not determined due to minimal oil output.

**Table 3:** Determined parameters of the pressed oil at 14 days in storage under laboratory conditions.

Oilseeds	$M_{RS}$ (g)	$M_{OL}$ (g) ***	$M_{RS}$ (%)	$O_{YD}$ (%)	$M_{SK}$ (%)	$M_{ML}$ (%)
Sesame	11.60 ± 0.40	59.89 ± 3.59	5.80 ± 0.20	29.95 ± 1.80	60.99 ± 0.04	3.27 ± 1.56
Pumpkin	9.98 ± 0.28	26.33 ± 0.54	4.99 ± 0.14	13.17 ± 0.27	78.38 ± 0.07	3.47 ± 0.21
Flax	5.16 ± 0.64	54.42 ± 1.10	2.58 ± 0.32	27.21 ± 0.55	68.69 ± 0.37	1.53 ± 0.50
Milk thistle	5.62 ± 1.32	28.02 ± 0.94	2.81 ± 0.66	14.01 ± 0.47	80.77 ± 0.13	2.42 ± 1.00
Hemp	6.11 ± 0.54	40.08 ± 0.21	3.06 ± 0.27	20.04 ± 0.11	73.69 ± 0.07	3.22 ± 0.10
Cumin	****	****	****	****	94.11 ± 0.07	2.43 ± 0.07

$M_{RS}$: mass of seedcake residues/sediments in oil; $M_{OL}$: mass of extracted crude oil; $O_{YD}$: oil yield; $M_{SK}$: mass of seedcake; $M_{ML}$: percentage material losses; ***: without seedcake residues/sediments in the oil. ****: not determined due to minimal oil output.

**Table 4:** Determined parameters of the pressed oil at 21 days in storage under laboratory conditions.

Oilseeds	$M_{RS}$ (g)	$M_{OL}$ (g) ***	$M_{RS}$ (%)	$O_{YD}$ (%)	$M_{SK}$ (%)	$M_{ML}$ (%)
Sesame	10.30 ± 0.18	61.20 ± 3.37	5.15 ± 0.09	30.60 ± 1.69	60.99 ± 0.04	3.27 ± 1.56
Pumpkin	9.56 ± 0.44	26.75 ± 0.70	4.78 ± 0.22	13.37 ± 0.35	78.38 ± 0.07	3.47 ± 0.21
Flax	4.12 ± 0.70	55.46 ± 1.04	2.06 ± 0.35	27.73 ± 0.52	68.69 ± 0.37	1.53 ± 0.50
Milk thistle	4.71 ± 1.23	28.93 ± 1.03	2.36 ± 0.62	14.46 ± 0.51	80.77 ± 0.13	2.42 ± 1.00
Hemp	5.57 ± 0.55	40.63 ± 0.22	2.79 ± 0.28	20.31 ± 0.11	73.69 ± 0.07	3.22 ± 0.10
Cumin	****	****	****	****	94.11 ± 0.07	2.43 ± 0.07

$M_{RS}$: mass of seedcake residues/sediments in oil; $M_{OL}$: mass of extracted crude oil; $O_{YD}$: oil yield; $M_{SK}$: mass of seedcake; $M_{ML}$: percentage material losses; ***: without seedcake residues/sediments in the oil. ****: not determined due to minimal oil output.

Fourthly, the multivariate test of significance of the effect of the processing factors (oilseed type and storage duration) on oil yield and sediments in the oil as well as the one-way ANOVA test of significance of the effect of oilseed type on the mass of seedcake and percentage material losses are presented in Table 5 and Table 6. The types of oilseeds and storage duration of the extracted crude oil significantly (*p*-value < 0.05) influenced the oil yield percentage and sediments in the oil. This implies that oil content varies in different oilseeds. Seedcake sediments in the extracted crude oil decreased over time; thus, cleaned or refined oil can be obtained or recovered, which subsequently increases oil yield. However, the effect of interaction between oilseed type and storage duration was not significant (*p*-value > 0.05). Additionally, based on the one-way ANOVA results, oilseed type significantly (*p*-value < 0.05) increased the mass of seedcake but did not significantly affect (*p*-value > 0.05) the material losses (oil and sediments). Shapiro–Wilk’s test of normality was used to test the normality of the data [95]. It follows that the data were normally distributed (*p*-value > 0.05) as shown in Figure 6.

**Table 5:** ANOVA multivariate test of significance of the effect parameters on oil yield and sediments.

Effect	Wilks Value	*F*-Value	Effect df	Error df	*p*-Value
Oilseed type	<0.05	203.98	8	28	<0.05
Storage duration	<0.05	29.49	4	28	<0.05
Interactions	>0.05	0.65	16	28	>0.05

df: degrees of freedom; *p*-value < 0.05 or larger *F*-value means significant and *p*-value > 0.05 means non-significant.

**Table 6:** Test of the sum of squares whole model versus residual of the dependent parameters against oilseed type.

Dependent Parameters	R^2^	*F*-Value	*p*-Value
$M_{SK}$ (%)	0.99	9067.51	<0.05
$M_{ML}$ (%)	0.59	1.73	>0.05

$M_{SK}$: mass of seedcake; $M_{ML}$: percentage material losses; *p*-value < 0.05 or larger *F*-value means significant and *p*-value > 0.05 means non-significant.

Lastly, the obtained cleaned oils without any seedcake sediments were evaluated for some quality parameters (peroxide value, *PV*; acid value, *AV*; free fatty acid, *FFA* and iodine value, *IV*) as shown in Table 7. *PV* values ranged from 2.37 ± 0.67 to 18.45 ± 0.53 meq O_2_/kg oil, *AV* values ranged from 2.83 ± 0.08 to 11.21 ± 0.24 mg KOH/g oil, *FFA* values ranged from 1.20 ± 0.04 to 5.60 ± 0.12 mg KOH/g oil, and *IV* values ranged from 13.61 ± 0.07 to 14.53 ± 0.82 g l/100 g. It was observed from the data that pumpkin oil had the highest *PV*, *AV* and *FFA* values but showed the second-highest *IV* values after the flax oil sample. The *IV* values for the oil samples did not vary greatly compared to the other quality parameters. For *PV*, the lowest value at 2.37 ± 0.67mg KOH/g oil was recorded for the flax oil sample. Hemp oil showed the lowest *AV* value at 2.40 ± 0.08 mg KOH/g oil and *FFA* of 1.20 ± 0.04 mg KOH/g oil. Table 8 presents the results of the univariate ANOVA of the determined oil quality parameters against the oilseed type. *PV*, *AV* and *FFA* were statistically significant (*p*-value < 0.05) except for iodine value (*IV*) which was found not to be statistically significant (*p*-value > 0.05). The coefficient of determination (R^2^) values ranged between 0.804 and 0.999. This implies that the determined quality parameters varied with oilseed type apart from *IV* which was significantly homogeneous (*p*-value < 0.05). In general, the peroxide value (*PV*) measures the extent to which rancidity reactions have occurred during storage, which is used as an indication of the quality and stability of fats and oils [5,96,97]. Acid value (*AV*) is a measure of the degree of decomposition of the oil via the action of lipases or other causes, which is accelerated by light and heat [5,98]. The iodine value (*IV*) measures the degree of unsaturation in a fat or vegetable oil, which determines the stability of the oxidation of oils. The oxidative and chemical changes in oils during storage are characterized by an increase in free fatty acid content and a decrease in the total unsaturation of oils [5,97,99]. It is important to mention that the storage duration of the oil samples, among other factors such as temperature and contact with the air, could influence the quality parameters discussed herein [100]. This analysis would be conducted in future studies. Figure 7 compares the quality parameters of the cold-pressed oils from the selected oilseeds.

**Table 7:** Chemical properties of pressed oils after 1 week storage under laboratory conditions.

Bulk Oilseeds (Pressed Oils)	*PV* (meq O_2_/kg Oil)	*AV* (mg KOH/g Oil)	*FFA* (mg KOH/g Oil)	*IV* (g l/100 g)
Sesame	4.77 ± 0.01	2.83 ± 0.08	1.41 ± 0.04	14.44 ± 0.24
Pumpkin	18.45 ± 0.53	11.21 ± 0.24	5.60 ± 0.12	14.49 ± 0.16
Flax	2.37 ± 0.67	2.85 ± 0.04	1.43 ± 0.02	14.53 ± 0.82
Milk thistle	10.82 ± 0.59	5.27 ± 0.16	2.63 ± 0.08	13.88 ± 0.11
Hemp	9.00 ± 0.69	2.40 ± 0.08	1.20 ± 0.04	13.61 ± 0.07
Cumin	****	****	****	****

PV: peroxide value; AV: acid value: FFA: free fatty acid; IV: iodine value; ****: no test conducted due to minimal oil output.

**Table 8:** Univariate results for the determined chemical properties against oilseed type.

*PV*, Peroxide Value (meq O_2_/kg Oil) ^a^					
Effect	df	Sum of squares	Mean square	*F*-Value	*p-Value*
Oilseeds	4	309.033	77.258	249.211	<0.05
Error	5	1.5501	0.3100		
Total	9	310.583			
***AV*, Acid value (mg KOH/g oil)** ^b^					
**Effect**	**df**	**Sum of squares**	**Mean square**	***F*-value**	***p-value***
Oilseeds	4	109.3995	27.349	1439.58	<0.05
Error	5	0.095	0.019		
Total	9	109.495			
***FFA*, Free fatty acid (mg KOH/g oil)** ^c^					
**Effect**	**df**	**Sum of squares**	**Mean square**	***F*-value**	***p-value***
Oilseeds	4	27.34987	6.837	1439.58	<0.05
Error	5	0.024	0.005		
Total	9	27.374			
***IV*, Iodine value (g l/100 g)** ^d^					
**Effect**	**df**	**Sum of squares**	**Mean square**	***F*-value**	***p-value***
Oilseeds	4	1.399	0.350	2.28	>0.05
Error	5	0.767	0.153		
Total	9	2.166			

df: degrees of freedom; *p*-value < 0.05 or larger F-value means significant and *p*-value > 0.05 means non-significant; ^a^, ^b^, ^c^ and ^d^: coefficient of determination (R^2^) = 0.995, 0.999, 0.999 and 0.804.

## 5. Conclusions

Sesame, pumpkin, flax, milk thistle, hemp and cumin oilseeds were cold-pressed to determine the amount of crude oil, seedcake and material losses (oil and sediments) using a small screw press. Sesame had the largest amount of crude oil of 71.49 ± 3.20 g, whereas cumin seeds produced the smallest amount of crude oil of 6.91 ± 0.29 g. The amounts of crude oil from flax, hemp, pumpkin and milk thistle ranged from 59.57 ± 1.74 to 33.64 ± 2.26 g. It was observed that the lower the amount of crude oil extracted, the higher the seedcake produced. The percentage of material losses during the pressing operation ranged from 1.53 ± 0.50% to 3.27 ± 1.56%, indicating high efficiency of the oil press. Cumin oilseed was found not suitable for processing under screw-pressing operation due to its minimal oil output. Oilseed type and storage duration had a significant positive effect (*p*-value < 0.05) on the percentage oil yield, which ranged from 30.60 ± 1.69% to 13.37 ± 0.35% and seedcake sediments in the oil ranging from 5.15 ± 0.09% to 2.06 ± 0.35%, implying that storage duration thus enhances percentage oil yield with a smaller amount of seedcake sediments in the oil. Oilseed type had no significant effect on the material losses, indicating also that a considerable amount of the crude oil was extracted from the selected oilseeds. For the 7-day storage duration, peroxide value, acid value and free fatty acid except for iodine value significantly varied among the oilseed types. Peroxide values ranged from 2.37 ± 0.67 to 18.45 ± 0.53 meq O_2_/kg oil, acid values ranged from 2.83 ± 0.08 to 11.21 ± 0.24 mg KOH/g oil, free fatty acid values ranged from 1.20 ± 0.04 to 5.60 ± 0.12 mg KOH/g oil, and iodine values ranged from 13.61 ± 0.07 to 14.53 ± 0.82 g l/100 g. The oil yield increment of 3.47% and a 2.82% reduction of of the seedcake sediments in the oil were observed during storage. In future studies, there is a need to evaluate the economic benefits of the storage duration of crude vegetable oils in terms of energy requirement for obtaining high-quality refined oils with less or no seedcake sediments for domestic and industrial applications, analyze the oil recovery efficiency and specific energy requirement for cold-pressed oils using medium- to large-scale presses and examine the effect of storage duration on oil quality, sensory properties and oxidative stability of long term shelf-life of the refined oils. In addition, the temperature generation during the oil pressing operation should be monitored.

## Data Availability

The data presented in this study are available upon request from the corresponding author.
